# RLI-SLAM: Fast Robust Ranging-LiDAR-Inertial Tightly-Coupled Localization and Mapping

**DOI:** 10.3390/s24175672

**Published:** 2024-08-31

**Authors:** Rui Xin, Ningyan Guo, Xingyu Ma, Gang Liu, Zhiyong Feng

**Affiliations:** 1 Department of Information and Communication Engineering, Beijing University of Posts and Telecommunication, Beijing 100874, China; xinrui@bupt.edu.cn (R.X.); maxingyu@bupt.edu.cn (X.M.); fengzy@bupt.edu.cn (Z.F.); 2 Department of Electronic Engineering, Tsinghua University, Beijing 100084, China; liu_gang@tsinghua.edu.cn

**Keywords:** simultaneous localization and mapping, state estimation, loop closure detection, mapping, ultra-wideband

## Abstract

Simultaneous localization and mapping (SLAM) is an essential component for smart robot operations in unknown confined spaces such as indoors, tunnels and underground. This paper proposes a novel tightly-coupled ranging-LiDAR-inertial simultaneous localization and mapping framework, namely RLI-SLAM, which is designed to be high-accuracy, fast and robust in the long-term fast-motion scenario, and features two key innovations. The first one is tightly fusing the ultra-wideband (UWB) ranging and the inertial sensor to prevent the initial bias and long-term drift of the inertial sensor so that the point cloud distortion of the fast-moving LiDAR can be effectively compensated in real-time. This enables high-accuracy and robust state estimation in the long-term fast-motion scenario, even with a single ranging measurement. The second one is deploying an efficient loop closure detection module by using an incremental smoothing factor graph approach, which seamlessly integrates into the RLI-SLAM system, and enables high-precision mapping in a challenging environment. Extensive benchmark comparisons validate the superior accuracy of the proposed new state estimation and mapping framework over other state-of-the-art systems at a low computational complexity, even with a single ranging measurement and/or in a challenging environment.

## 1. Introduction

Simultaneous localization and mapping (SLAM) is an essential component for smart robot operations in unknown confined spaces such as indoors, tunnels and underground [1]. A variety of LiDAR-based SLAM systems have been widely employed due to their advantages of high resolution, robustness to low-light environments and dense 3D map ability [2,3,4,5]. Due to the rapid development of lightweight and cost-effective LiDAR technologies, LiDAR-based SLAM systems show great potential applications for small unmanned platforms with limited computation resources [6].

However, high-accuracy, fast and robust LiDAR odometry and mapping are encountered three main practical challenging problems [7]. The first one is the point cloud distortion caused by LiDAR’s fast motion, which introduces severe state error in the long-term scenario. The second one is geometric degeneration in the challenging environment such as strong-light spaces and straight tunnels, which cause mapping distortions. The third one is a large number of point clouds generated in real-time, which causes a processing load on limited onboard computing resources.

Sensor fusion is the most used approach to overcome the first two shortcomings of the LiDAR in the LiDAR-based system. It has been proven that integrating LiDAR and other sensors with complementary properties, such as the inertial sensors and cameras, can improve the state estimation accuracy [8,9]. However, most of these works do not solve the computational complexity problem of the LiDAR sensor. To reduce the computation load, Xu et al. [4] propose a direct method to register the raw points to estimate the state and build the map without extracting features, which achieves higher accuracy at a much lower computation load than other state-of-the-art LiDAR-inertial SLAM systems. However, they assume that the inertial sensor does not have any bias in the initial fusion phase and can compensate for the point cloud distortion of the moving LiDAR. But, once the inertial sensor has a bias, the inertial sensor cannot compensate for the point cloud distortion of the moving LiDAR, and then, inevitably causes the error state estimation. Therefore, when the state estimation is inaccurate, we cannot correct the bias and experience long-term drift and the whole SLAM system diverges. Moreover, they do not apply an effective loop closure detection module, which causes mapping distortion in a challenging environment.

Due to the centimeter-level ranging accuracy, high temporal resolution, and resistance to the multipath effect of the ultra-wideband (UWB) system [10,11], several works involve loosely coupling the position results generated from the UWB system into the LiDAR-based SLAM [12,13,14,15]. However, these works require a large number of UWB anchors as support to calibrate the bias of the system, which may lead to significant errors in position estimation in environments with poor UWB anchor distribution, thereby affecting the state estimation of the entire SLAM system.

In this paper, we propose a novel tightly-coupled ranging-LiDAR-inertial simultaneous localization and mapping framework, namely RLI-SLAM, designed to achieve high accuracy, speed, and robustness in the long-term fast-motion scenarios in sparsely or poorly anchored environments. The main contributions of this paper include the following:

- We tightly fuse the high-accuracy UWB ranging measurements with the inertial sensor, which can effectively eliminate the initial bias and long-term drift of the inertial sensor. This allows the point cloud distortions of the fast-moving LiDAR to be effectively compensated in real-time, whether in the initial phase or the long-term processing, even with a single anchor’s ranging measurement.
- We introduce an efficient loop closure detection module at a low computational complexity, utilizing an incremental smoothing factor graph approach. This module seamlessly integrates into our RLI-SLAM system, enabling high-precision mapping in challenging environments.
- We conduct extensive benchmark comparisons and validate that, compared with other state-of-the-art systems, our approach is highly accurate, robust, flexible, and fast for state estimation and mapping in long-term fast-motion scenarios. Specifically, there is no limitation on the number of tightly-coupled ranging measurements, and we add an efficient loop closure detection module that can be seamlessly integrated into our RLI-SLAM system to improve accuracy. As for flexibility, even without ranging measurement, we can still use tightly-coupled LiDAR and inertial sensors to maintain the high-accuracy state estimation. Additionally, our approach has the same low computational complexity as the fast LiDAR-Inertial odometry (FAST-LIO2) [4] system, which is the fastest LiDAR-based odometry available.

## 2. Related Works

### 2.1. UWB-LiDAR-Inertial Odometry

In recent research, many researchers have attempted to incorporate UWB into LiDAR-inertial SLAM systems. For instance, in [13], a loosely coupled sensor fusion method is introduced to diminish LiDAR odometry drift by leveraging positioning data from two relatively independent systems. Conversely, tightly-coupled methods such as those discussed in [14] merge 2D LiDAR ranging with UWB measurements to mitigate cumulative errors in the LiDAR data. Although these approaches are effective, they necessitate a substantial quantity of UWB anchors to create a “coarse” map through ranging measurements that assist LiDAR in constructing a “fine” map. However, when the number of available UWB anchors is restricted, the state estimation and mapping could be inaccurate due to the excessively “coarse” map resulting from a limited number of UWB measurements. In recent research, [16] introduces a tightly-coupled sensor fusion method that utilizes factor graphs to incorporate UWB ranging into the SLAM system. This method can mitigate cumulative drift with only three anchors’ ranging values, yet its intricate computation results in a less significant enhancement of the state estimation in accuracy.

### 2.2. Loop-Closure Detection

In a SLAM system, the primary goal of the loop closure correction is to identify loops in the robot’s trajectory and rectify them, mitigating the odometry drift caused by noise, environmental variations, and sensor errors. As shown in [17,18,19], the local key points voting method is adopted to carry out sub-linear matching for loop closure detection. Moreover, different from the local key points method, such as multiview 2D projection (M2DP) [20], global key descriptors utilize LiDAR scan points, which are known for their resilience to noisy input. However, these methods encounter challenges during seamless integration into any LiDAR system, which constrains their applicability.

## 3. System Architecture

**Figure 1:**
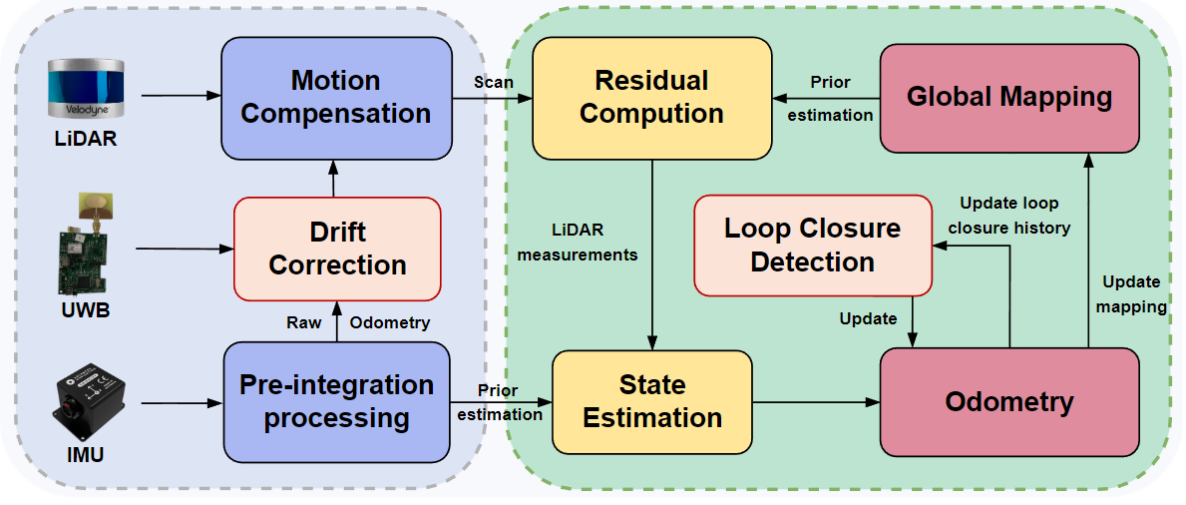
System overview.

As described in Figure 1, the front-end of our RLI-SLAM system takes LiDAR point cloud data, UWB ranging measurements, and the inertial sensor data as input to estimate the prior state. After synchronizing the sensors’ data, the UWB ranging is tightly fused with the inertial sensor to prevent the initial bias and long-term drift of the inertial sensor. This allows the inertial sensor to undergo pre-integration processing and provide a prior state estimation to effectively compensate for the point cloud distortion of the fast-moving LiDAR in real-time. In the back-end, new scan points from the LiDAR are combined with the prior estimation to perform the state estimation through using an iterative error state Kalman filter (IESKF) and registered into an incremental k-d tree (ik-d tree) data structure to efficiently build a dense map. The resulting loop closure detection measurements are combined with the state estimation to provide odometry, and to update the global dense map.

## 4. Methodology

Our system employs inertial sensor measurements and LiDAR measurements as observations and utilizes IESKF for data fusion. To mitigate the inertial sensor’s initial bias and long-term drift, as well as to compensate for point cloud distortion caused by the high-speed LiDAR, we utilize UWB-ranging data to aid LiDAR motion compensation with minimal computational complexity. Additionally, we have integrated loop closure detection to achieve high-precision mapping in challenging environments, thereby enhancing system robustness and accuracy. Detailed explanations will be provided in the following sections.

### 4.1. Preliminaries

#### 4.1.1. State Estimates

By utilizing the inertial sensor coordinate system as the body reference coordinate system and defining its initial frame as the global coordinate system, we can derive the kinematic model in the global coordinate system.

(1) $$x={\left[p^{T},v^{T},\theta^{T},b_{\alpha }^{T},b_{\omega }^{T},g^{T}\right]}^{T},$$

where x represents the state variable that varies over time, p, v, and θ represent the inertial sensor’s displacement, velocity, and the Euler angles, respectively, $b_{\omega }$ and $b_{\alpha }$ represent the biases of the inertial sensor’s angular and acceleration, respectively, while g represents the unknown gravity vector in the kinematic model.

We denote the continuous-time accelerations and angular velocities read from the inertial sensor as $\hat{\alpha }$ and $\hat{\omega }$, respectively, and express the relationship between the derivatives of the corresponding error state variables and the observations in the continuous kinematic model as

$$\delta \dot{p}=\delta v,\delta {\dot{b}}_{\alpha }=\eta_{b\alpha },\delta {\dot{b}}_{\omega }=\eta_{b\omega },\delta g=0$$

$$\delta \dot{v}=-R{\left(\hat{\alpha }-b_{\alpha }\right)}^{\wedge }\delta \theta -R\delta b_{\alpha }-\eta_{\alpha }+\delta g$$

(2) $$\delta \dot{\theta }=-{\left(\hat{\omega }-b_{\omega }\right)}^{\wedge }\delta \theta -\delta b_{\omega }-\eta_{\omega },$$

where ${\left(a\right)}^{\wedge }$ represents the skew-symmetric matrix of a vector $a\in R^{3}$, R is the direction cosine matrix of θ, $\eta_{\alpha }$ and $\eta_{\omega }$ represent the white noise of the inertial sensor-measured acceleration and angular velocity, while $b_{\alpha }$ and $b_{\omega }$ represent the bias for acceleration and angular velocity, respectively, which are modeled as Gaussian noise and follow a random walk process characterized by $\eta_{b\alpha }$ and $\eta_{b\omega }$. The discrete motion model derived from Equation (2) using the sampling period Δt of the inertial sensor is

$$\delta p\left(t+\Delta t\right)=\delta p\left(t\right)+\delta v\Delta t,\;\delta b_{\alpha }\left(t+\Delta t\right)=\delta b_{\alpha }\left(t\right)+\eta_{b\alpha }$$

$$\delta b_{\omega }\left(t+\Delta t\right)=\delta b_{\omega }\left(t\right)+\eta_{b\omega },\;\delta g\left(t+\Delta t\right)=\delta g\left(t\right)$$

$$\delta v\left(t+\Delta t\right)=\delta v\left(t\right)+\left(-R{\left(\hat{\alpha }-b_{\alpha }\right)}^{\wedge }\delta \theta -R\delta b_{\alpha }+\delta g\right)\Delta t-\eta_{v}$$

(3) $$\delta \theta \left(t+\Delta t\right)=exp\left(-\left(\hat{\omega }-b_{\omega }\right)\Delta t\right)\delta \theta \left(t\right)-\delta b_{\omega }\Delta t-\eta_{\theta },$$

The discrete-time state of speed is derived from the time derivative part of speed in Equation (2). The rotational part can be obtained using the integral formula of angular velocity. Specifically, by treating the time derivative part of angular velocity in Equation (2) as a differential equation with respect to δθ and solving it, we can obtain the integral related to the rotational part.

#### 4.1.2. Synchronization

**Figure 2:**
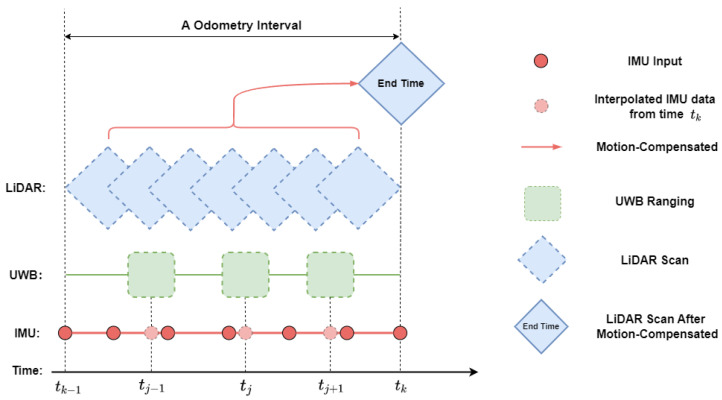
Synchronization among the sensors.

The time synchronization of sensors within the system has been adapted from the time synchronization principles documented in [10], with a focus on LiDAR and IMU. Given the relatively lower frequency of UWB, it often necessitates the interpolation and adaptation of its data using the higher frequency IMU within scanning intervals, as depicted in Figure 2.

### 4.2. UWB-LiDAR-Inertial Odometry

#### 4.2.1. Motion Compensation

Our system utilizes LiDAR sensor data as observation. To effectively utilize the high frequency of the inertial sensor data, we employ the inertial sensor measurements to estimate the relative pose of each LiDAR point at the end of the scan. This compensation effectively mitigates motion offsets of the LiDAR sensor, ensuring swift and accurate observations for our system’s state propagation and enhancing the system’s robustness.

The estimated rough attitude provided by the inertial sensor allows us to project points from each sampling moment in the LiDAR scan to align with the moment when the scan concludes. As a result, all points from every LiDAR scan are considered as points at the moment when the scan concludes. The process of motion compensation for LiDAR is as follows:

(4) $${\tilde{P}}_{L}={R_{L}^{I}}^{-1}\left(R_{I}^{-1}\left(R_{I}^{G}\left(R_{L}^{I}\cdot P_{L}+P_{L}^{I}\right)+\Delta T_{I}\right)-P_{L}^{I}\right),$$

where $P_{L}^{I}$ and $R_{L}^{I}$ represent the translation and rotation of the rigidly connected LiDAR to the inertial sensor, $P_{L}$ refers to a series of poses of LiDAR in the laser coordinate system before motion compensation, $R_{I}^{G}$ is the rotation matrix from the inertial sensor coordinate system to the global coordinate system (the coordinates of the first frame of the inertial sensor coordinate system), $\Delta T_{I}$ is the translation from the position of the inertial sensor in the global coordinate system at the end of the scan to the current inertial sensor point position, $R_{I}^{-1}$ is the inverse of the rotation matrix from the inertial sensor output, and ${\tilde{P}}_{L}$ represents a series of poses of LiDAR in the LiDAR coordinate system after motion compensation and distortion correction.

#### 4.2.2. UWB Constraint and Drift Correction

The key challenge in motion compensation within the LIO (Lidar Inertial Odometry) system lies in accurately projecting lidar points from one scan to the latest pose, which is the attitude after IMU preintegration. This challenge is closely tied to the quality of the corresponding attitudes of lidar points at different measurement times. While the state remains consistent within the IESKF system composed of IMU and LiDAR, short-term propagation should be accurate. However, both vibrations and movements of the body can cause temporary offsets in LiDAR, thereby diminishing the quality of corresponding attitudes at different measurement times and leading to subsequent sustained drift in the system. Hence, we introduce UWB ranging measurements between UWB nodes and anchors to augment and fine-tune the initial attitude estimation derived from the inertial sensor. This serves to strengthen the process of projecting lidar points from one scan to the latest pose, ultimately enhancing the accuracy and robustness of the system. It is worth noting that the world coordinate system in our system aligns with the initial keyframe’s pose. We can obtain the coordinates of the anchors in the anchor coordinate system by measuring the relative distances between the anchors and designating one of the anchors as the origin. By applying a common transformation from the UWB anchor coordinate system to the world coordinate system, we can determine the coordinates of all anchors in the world coordinate system. We represent this transformation as follows:

(5) $$T_{U}^{W}=\left(R_{U}^{W},p_{U}^{W}\right),$$

where $R_{U}^{W}$ and $p_{U}^{W}$ represent the rotation and transformation of the rigidly connected UWB to the world, and this transformation can be easily estimated within the state propagation of the IESKF. The measurement state of UWB at time step *j* can be described as:

(6) $$U_{j}^{i}=\left(a_{i},b,t_{j},d_{i}\right),i=1,2,3,...,N,$$

where *i* is index of the UWB anchor points, $a_{i}$ represents the global coordinate system coordinates of the *i*-th UWB anchor point, b is the offset of the UWB node relative to the body in the body coordinate system, $t_{j}$ is the time of this measurement state in the system, $d_{i}$ is the distance measurement data between the UWB node and the *i*-th UWB anchor point. At time step *j*, the distance between the node and UWB anchor point *i* can be described using the rough pose estimation derived from the inertial sensor:

$$d\left(\chi_{k},\delta \theta \right)=p_{k}-\left(R_{U}^{W}a_{i}+p_{U}^{W}\right)$$

$$+R_{k-1}\exp\left(\delta \theta \right)exp\left(\Delta t_{1}\mathrm{Log}\frac{R_{k}\exp\left(\delta \theta \right)}{R_{k-1}\exp\left(\delta \theta \right)}\right)b$$

(7) $$-\frac{1}{2}\delta v\Delta t_{2}+\eta_{d},$$

where $\Delta t_{1}=\frac{t_{j}-t_{k-1}}{t_{k}-t_{k-1}},\Delta t_{2}=t_{j}-t_{k-1}$, $\chi_{k}$ represents the rough pose state estimated by the inertial sensor at time step *k*,δv and δθ is the rotational error state of the rough pose estimated by the inertial sensor in (3), $p_{k}$ is the rough coordinate estimated by the inertial sensor, $\eta_{d}$ is the noise. Therefore, the residual between the distance measurement value of the *i*-th UWB anchor point at time step *k* and its estimated value can be expressed as:

(8) $$r_{U}\left(\chi_{k},U_{k}^{i}\right)=∥d\left(\chi_{k},\delta \theta \right)∥-d_{i}.$$

This nonlinear optimization requires extremely low computational resources to quickly and accurately enhance the system’s precision. Furthermore, since it corrects the LiDAR motion compensation module rather than the entire system, only a small number of UWB anchor points are needed to achieve this precision improvement, as confirmed by subsequent benchmark dataset experiments.

#### 4.2.3. Observation Model

To obtain the observation equation, it is necessary to transform the motion-compensated ${\tilde{P}}_{L}$ from (4) into the global coordinate system:

(9) $$G_{{\tilde{P}}_{L}}=T_{L}^{G}{\tilde{P}}_{L}+\eta_{P_{L}},$$

where $T_{L}^{G}$ represents the transformation from the LiDAR coordinate system to the global coordinate system, ${\tilde{P}}_{L}$ denotes the noise after motion compensation, and the transformed $G_{\tilde{P_{L}}}$ should ideally lie on a local flat plane (or edge) within the global map. To achieve this, we employ a k-d tree search to find *n* nearest points $G_{P_{i}}$ for plane fitting. These points are fitted into a plane, and we assume $G_{P_{i}}$ to be the true position of $G_{\tilde{P_{L}}}$ in the global map. Thus, we can construct the residual:  

(10) $$r_{L}(\chi_{k},G_{\tilde{P_{L}}})=∥u^{T}\left(G_{\tilde{P_{L}}}-G_{P_{i}}\right)∥,$$

where *u* represents the normal vector of the fitted plane.

Multiple LiDAR data obtained through motion compensation can be iteratively incorporated into our system to obtain the solution and propagate the state.

### 4.3. Loop Closure Detection

**Figure 3:**
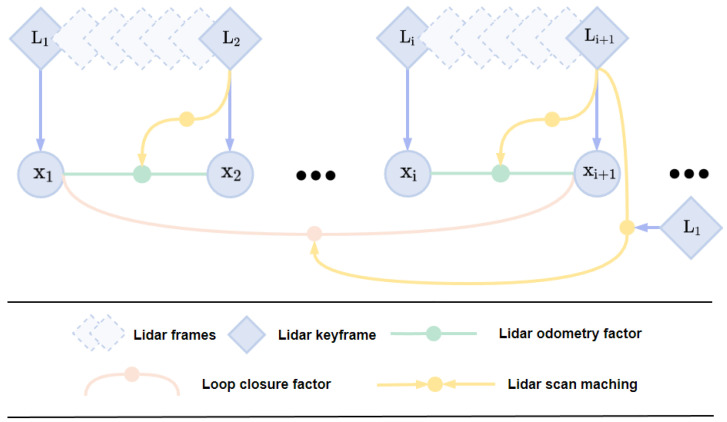
Loop detection module factor graph.

We aim to utilize backend loop closure detection to correct the robot’s state and its trajectory. The state estimation problem can be formulated as a Maximum A Posteriori (MAP) problem. We employ factor graphs to model this problem, as they are more suitable for inference compared to Bayesian networks. To construct the factor graph, we introduce two types of factors and one variable type. The variable represents the state of the robot at a specific time and is attributed to nodes in the graph. The two types of factors are odometry factors and loop closure factors. When the change in the robot’s pose exceeds a user-defined threshold, a new robot state node *x* is added to the graph. Utilizing incremental smoothing and mapping (iSAM2), the factor graph is optimized upon the insertion of new nodes (depicted in Figure 3). Consequently, our proposed loop closure detection module can seamlessly integrate into any odometry system. The following sections describe the process of generating these factors.

#### 4.3.1. Odometry Factor

The computational challenge of calculating and adding factors to the graph for every odometry frame is significant. To address this, we employ the concept of keyframe selection, a strategy widely used in the field of visual Simultaneous Localization and Mapping (SLAM). We use a straightforward yet effective heuristic approach, selecting the odometry frame $L_{i}$ as a keyframe when the change in the robot’s pose surpasses a user-defined threshold relative to the previous state $x_{i}$. The newly stored keyframe $L_{i+1}$ is then linked with the new robot state node $x_{i+1}$ in the factor graph. Any odometry frames that occur between two keyframes are disregarded. This method of adding keyframes not only strikes a balance between map density and memory usage but also helps to maintain a relatively sparse factor graph, which is conducive to real-time nonlinear optimization. In our research, we have set the thresholds for selecting position and rotation changes for the addition of new keyframes at 1 m and 10 degrees, respectively.

Suppose we wish to add a new state node $x_{i+1}$ to the factor graph. The odometry keyframe associated with this state is $L_{i+1}$. To reduce computational complexity, we only input the pose transformation information from the frontend’s keyframe into the node $x_{i+1}$. This yields the relative transformation $\Delta T_{i,i+1}$ between $x_{i}$ and $x_{i+1}$, i.e., the odometry factor connecting these two pose states:

(11) $$\Delta T_{i,i+1}=T_{i}^{T}T_{i+1}+\delta ,$$

where $T_{i}\in SE\left(3\right)$ represents the *i*-th pose transformation in the world coordinate system, and δ denotes the noise term following a Gaussian distribution.

#### 4.3.2. Loop Closure Factor

Due to the utilization of factor graphs, unlike other laser-based loop closure detections, the closures designed by us can seamlessly integrate into the odometer systems of nearly all the laser scanners. For illustrative purposes, we devised and implemented a straightforward yet effective joint loop closure detection method based on Euclidean distance and the generation of point cloud descriptors refer to Algorithm 1, specifically utilizing the Scan Context descriptor (SCD) [21].

The Euclidean distance-based method does not transform the keyframes from the laser scanner into descriptors as mentioned above. Instead, each newly added keyframe is inserted into a k-d tree. By setting a predefined search radius and time interval, the method searches for the indices of neighboring points within the k-d tree, thus obtaining a set of neighboring points within the radius. This initial estimation is rough and requires subsequent refinement for higher precision localization, such as using the Iterative Closest Point (ICP) algorithm, as summarized in Algorithm 2.
**Algorithm 1:**  Loop Closure Detection Based on Point Cloud Descriptors    **Input**:  $ID_{cur}$: The index of the current keyframe                *L*: The set of loop closure frame indices                *d*: Loop closure search radius                *T*: Loop closure search time difference threshold

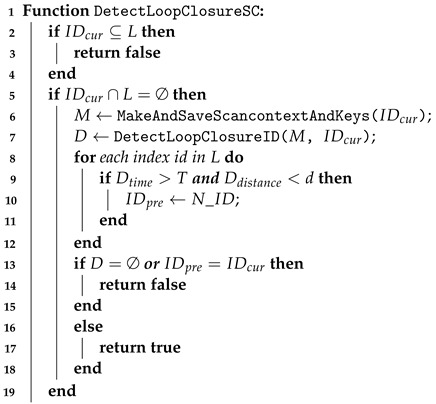


**Algorithm 2:**  Loop Closure Validity Check    **Input**: Index of the current keyframe $ID_{cur}$                Index of the loop closure keyframe $ID_{pre}$                Loop detection similarity threshold *S* 
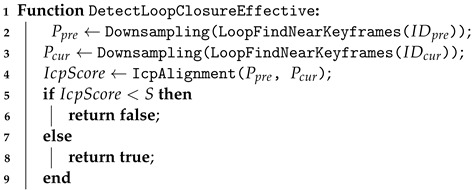


    It is noteworthy that in previous k-d tree-based proximity search systems, complete maps or submaps were used for search and matching [5]. In our system, however, lidar scans are employed as matching objects, corresponding to the observed objects in lidar-based state estimation. This approach not only maintains accuracy but also significantly improves matching speed. Although the Euclidean distance-based method is straightforward and effective, it may degrade when dealing with high-dimensional data, uneven data distribution, or significant outliers. Therefore, our system additionally incorporates a point cloud descriptor-based method refer to Algorithm 3). This method describes keyframe point clouds using an innovative spatial descriptor known as the SCD. The process begins by partitioning the raw measurements and using a bird’s-eye view (BEV) to project them into discrete cells. The proximity between two locations is then defined by the similarity score of the corresponding SCDs. If two SCDs are obtained from the same location, the descriptors should contain consistent content within a matrix, although there may be differences in column order.
**Algorithm 3:**  Loop Closure Detection Based on SCD    **Input**:  Index of the current keyframe $ID_{cur}$                 The set of loop closure frame indices *L*                 Loop closure search radius *d*                 Loop closure search time difference threshold *T*

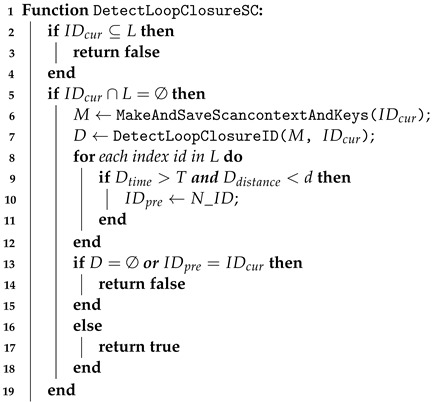


To measure similarity, we use cosine similarity between the two descriptors, which is particularly effective for dynamic objects or in the presence of partial noise. The cosine distance is used to calculate the distance between two column vectors $l_{c}^{j}$ and $l_{p}^{j}$ in the same column. The distance between the two descriptors is as follows:

(12) $$d\left(f_{p},f_{c}\right)=\frac{1}{N_{A}}\sum_{j=1}^{N_{A}}\left(1-\frac{l_{c}^{j}\cdot l_{p}^{j}}{∥l_{c}^{j}∥\cdot ∥l_{p}^{j}∥}\right)$$

where the subscripts *c* and *p* denote the current and past positions, where the descriptor has dimensions $f\in R^{N_{R}\times N_{A}}$, with $N_{A}$ representing the number of columns and $N_{R}$ representing the number of rows.

Combining these two loop detection methods allows for swift and adaptable correction of system drift in long-distance scenarios while utilizing minimal computational resources. This ultimately enhances system accuracy and robustness.

## 5. Experiments and Result

### 5.1. Benchmark Dataset

The dataset we utilize, known as M2DGR [22], was collected within the campus of SJTU and comprises multiple sequences recorded by a ground robot vehicle. This dataset features a Velodyne VLP-32C LiDAR sensor with a frequency of 10 Hz and a Handsfree A9 nine-axis inertial sensor with a frequency of 150 Hz. It encompasses various scenes within the campus environment, including structural buildings, lawns, lakes, and so forth. The second dataset is sourced from NTU’s campus, referred to as NTU VIRAL [23], collected using the Ouster OS1-16 first-generation LiDAR sensor at a scanning rate of 10 Hz. Gyroscope and accelerometer measurements are sampled at 385 Hz using a six-axis VN100 IMU. Data were recorded within the university campus, encompassing both indoor and outdoor locations, with sensor data captured by drones.

#### 5.1.1. UWB Anchors Configuration

**Figure 4:**
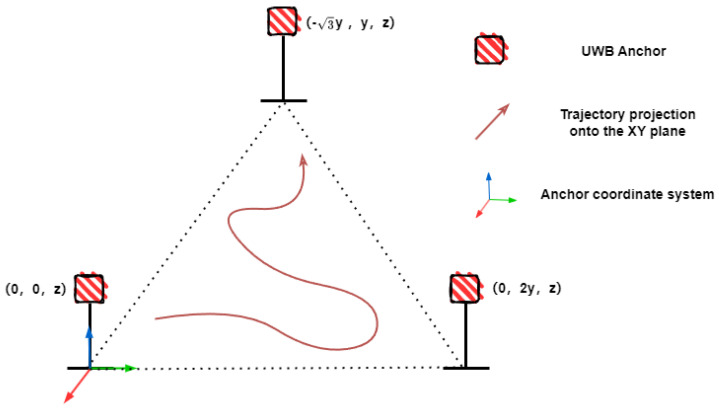
Anchor Coordinate Configuration.

Due to the lack of standardized UWB ranging datasets, in M2DGR, we simulated UWB ranging information by adding Gaussian noise with a mean of zero and a standard deviation of 5cm to the ground truth ranging data [24,25]. In our subsequent real-world experiments, the UWB noise is also modeled as Gaussian noise with a mean of zero and a standard deviation of 5 cm. For calculating the coordinates of anchors in the anchor coordinate system, as shown in Figure 4, it only requires setting one anchor as the origin and another anchor on the y-axis. The coordinates of the third anchor in the anchor coordinate system can be calculated based on the relative distances between the three anchors. This design allows for the straightforward calculation of anchor coordinates within the anchor coordinate system. By using the IESKF to estimate the transformation from the world coordinate system to the anchor coordinate system, the coordinates of the anchors in the world coordinate system can be accurately and easily determined.

#### 5.1.2. Accuracy Evaluation

In this section, we compare our system, RLI-SLAM, with other state-of-the-art LiDAR-based inertial odometry and mapping systems, including adaptive LiDAR odometry and mapping (A-LOAM) [3], LiDAR inertial odometry via smoothing and mapping (LIO-SAM) [5], and FAST-LIO2 [4]. In the M2DGR dataset, sequences *hall_01* and *hall_02* were obtained indoors by ground robots, while *door_01* and *door_02* depict transitions from indoor to outdoor environments, *and street_08* was collected during outdoor navigation. The ATE in the table clearly demonstrates our system’s consistent superiority in accuracy.

**Figure 5:**
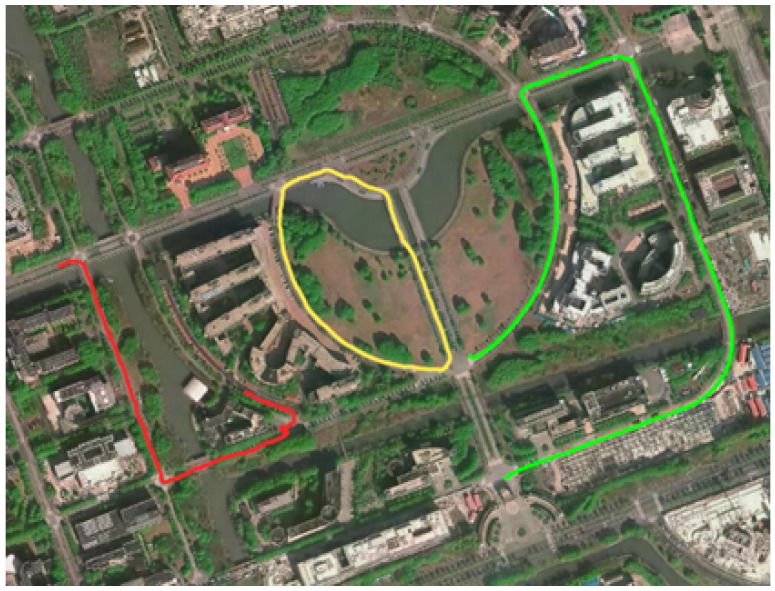
Trajectories of large-scale scene sequences on the map; with red representing the *street_01* sequence, green representing the *street_02* sequence, and yellow representing the *street_04* sequence.

For comprehensive experimentation, we included large-scale scene experiments, involving three sequences with long-distance trajectories, namely *street_01*, *street_02*, and *street_04*, as shown in Figure 5.

**Figure 6:**
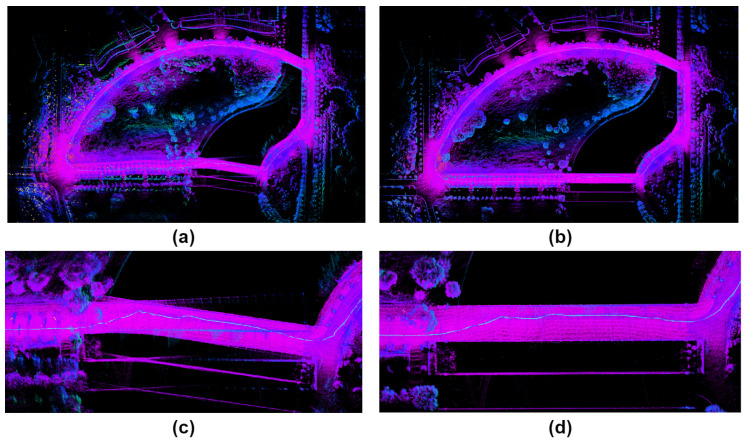
In the *street_04* sequence, (**a**,**c**) represent the global map constructed by FAST-LIO2 and the local map of its distorted portion, (**b**,**d**) depict the global map constructed by our proposed system and the corrected local map.

The data reveal significant drift in the *street_01* sequence for LIO-SAM, attributable to back-end factor graph optimization difficulties when handling prolonged and extensive data. Likewise, due to motion compensation drift in FAST-LIO2’s LiDAR, errors in residual estimation between planes and points lead to substantial distortion. In *street_04*, A-LOAM and FAST-LIO2, which lack loop closure detection modules, both exhibit poor accuracy due to cumulative drift over extended durations. The distorted global map generated by FAST-LIO2 is shown in Figure 6a, while the global map after correction by our system is depicted in Figure 6b. Although LIO-SAM showcases superior accuracy among the three methods for comparison, our system, leveraging both ranging and loop closure constraints concurrently, achieves the highest level of accuracy.

We conducted an assessment of UWB anchor point fusion quantities in RLI-SLAM, and examined how varying the number of fused UWB anchor points affects estimation drift in our system. We limited the number of fused ranging data to 1, 2, and 3 UWB anchor points. Additionally, an evaluation was conducted where the number of UWB anchor points received by the robot for ranging data was randomized between 0 and 3 to simulate real-world scenarios. Table 1 demonstrates that an increase in the number of fused UWB anchor points leads to a gradual improvement in overall accuracy. The result in the table shows that our system maintains high accuracy even with fewer UWB anchor points or randomly selected combinations.

We also conducted ablation experiments for loop closure detection. According to the data, it can be observed that in indoor environments such as *hall_01* and *hall_02*, where there are short-range, irregular movements in a confined space, loop closure detection introduces a certain negative impact on the overall system performance. However, in other scenes, particularly in longer trajectories such as *street_01*, *street_02*, and *street_04*, the removal of loop closure detection significantly affects the accuracy of our system.

**Figure 7:**
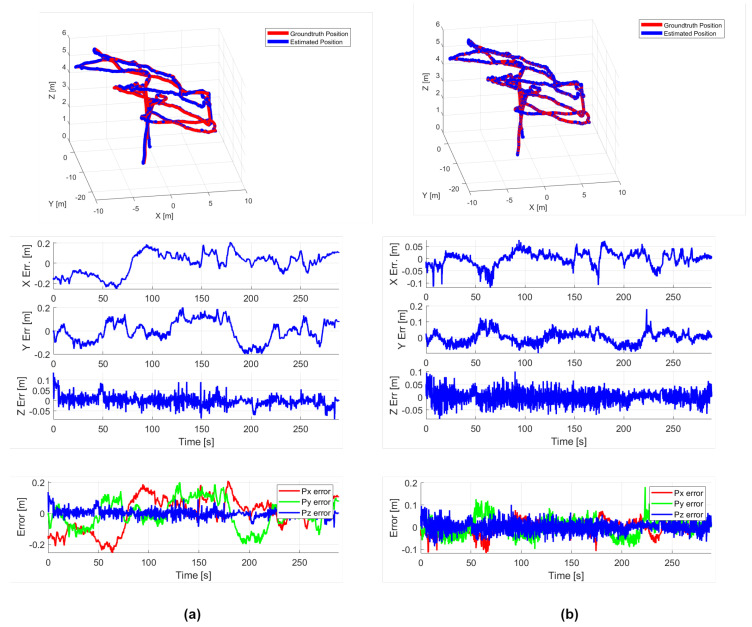
The result data from the *sbs_01* Sequence: (**a**) represents FAST-LIO2, (**b**) represents RIL-SLAM. From top to bottom, the figures are the trajectory plot, the individual error plots in the *x*, *y*, and *z* directions, and the combined error plot for *x*, *y*, and *z*.

In the experiments conducted on the NTU VIRAL dataset, we utilized three UWB anchors from the dataset as constraints, as shown in Table 2. The data are derived from the results presented in the VIRAL-Fusion [26] paper. VIRAL-Fusion employs two LiDARs, one camera, and three UWB anchors as constraints. As can be seen in Table 2, our method demonstrates higher accuracy performance. Additionally, our loop closure detection module further enhances the accuracy. Since VIRAL-Fusion is not open-source, we used FAST-LIO for comparison, as illustrated in Figure 7, it can be observed that our algorithm effectively suppresses drift compared to FAST-LIO2, significantly improving the overall pose accuracy.

#### 5.1.3. Processing Time Evaluation

To evaluate the computational efficiency of our system’s range constraint and loop detection components, we conducted module-specific timing experiments on a PC equipped with an Intel CPU E3-1275 v5.

Table 3 details the time consumption breakdown for scan processing. The data reveal that the UWB optimization section and the loop detection section each require 0.21 ms and 1.71 ms, respectively, constituting a small fraction of the total time. This suggests that these two computations minimally affect the overall computation time, highlighting our system’s high computational efficiency in these areas. Our system maintains a high computational efficiency for real-time localization and map building as a whole.

### 5.2. Real World Test

#### 5.2.1. Experimental Environment

**Figure 8:**
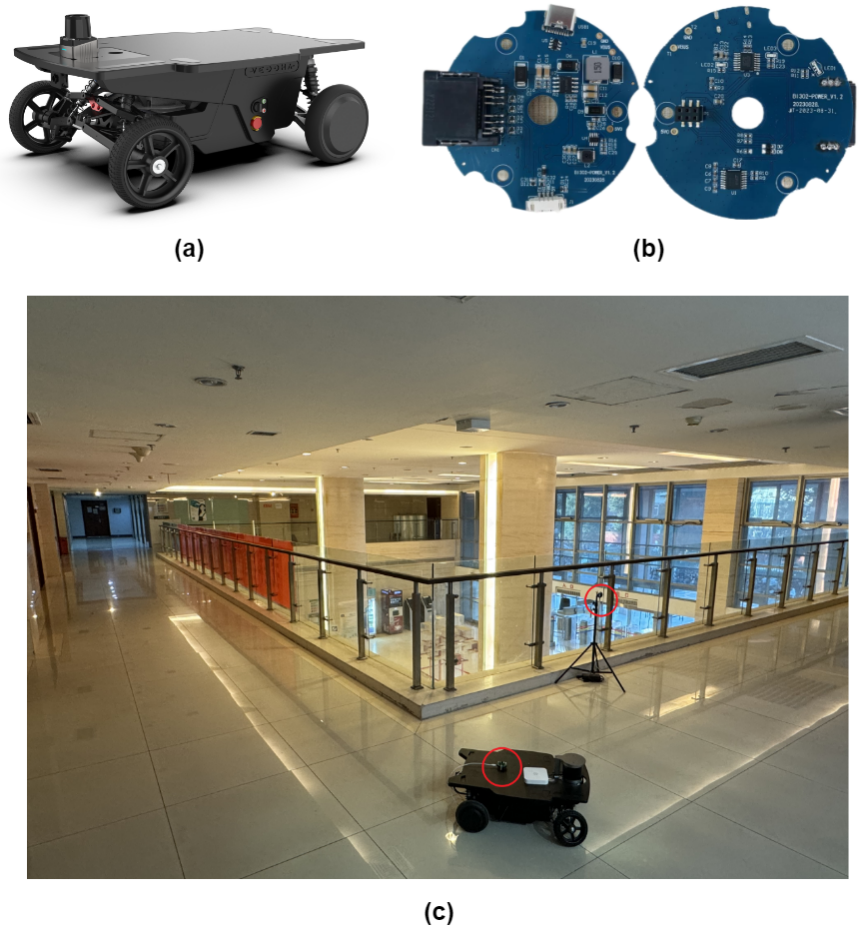
(**a**) Unmanned Vehicle Used in Experiment, (**b**) UWB Ranging Module, (**c**) Second Floor of BUPT Research Building with UWB Module Circled in Red.

As shown in Figure 8, the unmanned vehicle used in our experiment is equipped with a six-axis IMU operating at 200Hz and a 16-line Velodyne LiDAR operating at 20 Hz. Additionally, we utilized a UWB module with a ranging accuracy of 5cm and a maximum effective range of 100 m. The experiment was conducted on the second floor of the research building at BUPT. The space, characterized by a variety of textures and long corridor shapes, presented a challenging environment for LIO.

#### 5.2.2. Experimental Analysis

To avoid the influence of loop closure detection on the experiment, we only utilized our UWB calibration module in the real-world test, without activating the loop closure detection function. Additionally, to simplify the optimization of anchor coordinates transformation and further verify the robustness of our system to the number of anchors, only one anchor point was employed as the constraint for LiDAR motion compensation throughout the entire experimental process.

**Figure 9:**
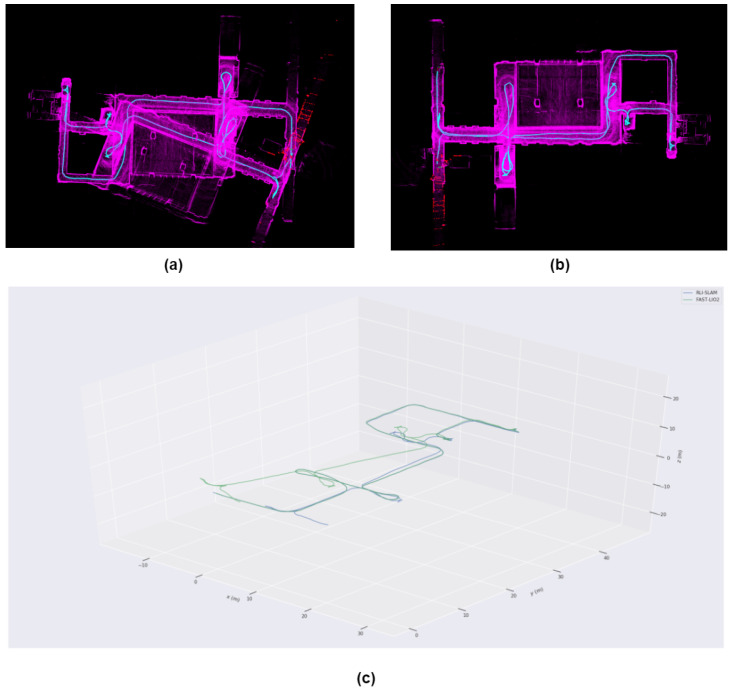
(**a**) Map constructed by FAST-LIO2 (**b**) Map constructed by RLI-SLAM (**c**) Trajectory plots of both methods.

**Figure 10:**
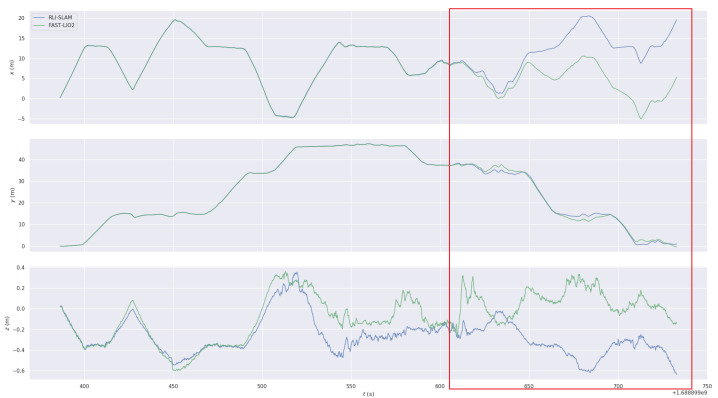
XYZ Components of Trajectory Estimation for FAST-LIO2 and RIL-SLAM.The red framed section indicates the part of the drift in FAST-LIO2.

From Figure 9 and Figure 10, it can be observed that FAST-LIO2 exhibits significant map distortion, whereas our system, calibrated solely by a single UWB anchor point, effectively corrected LiDAR motion drift in a timely manner, resulting in overall better performance.

**Figure 11:**
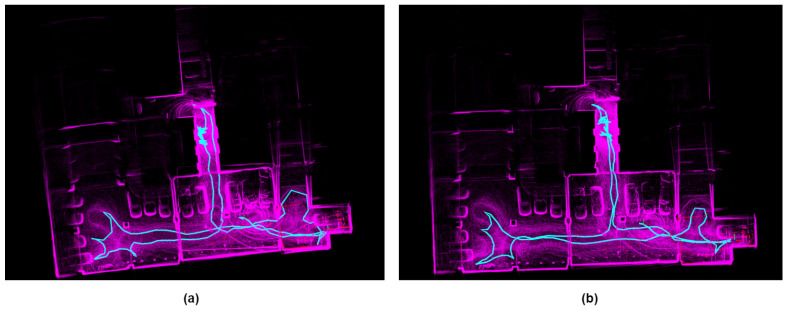
Experimental results from the basement.The (**a**) shows Fast-lio2, and the (**b**) shows our results.

To complement our experiments, we conducted a series of experiments in the underground parking environment as Figure 11. It was observed that while significant drift did not occur without UWB, the narrow and elongated layout caused by vehicles in the parking environment resulted in many minor drifts and overlaps in the map. Our method demonstrated greater robustness and accuracy in comparison.

## 6. Conclusions

In this paper, we introduce RLI-SLAM, a novel tightly-coupled ranging-LiDAR-inertial SLAM framework. By tightly fusing high-accuracy UWB ranging measurements with inertial sensor data, our framework effectively mitigates the distortion caused by fast-moving LiDAR, even with a single ranging measurement. We incorporate an efficient loop closure detection module using an incremental smoothing factor graph approach, ensuring high-precision mapping in challenging environments. Benchmark comparisons demonstrate the superior accuracy of our framework over state-of-the-art systems at low computational complexity. Our real-world experiments further validate the effectiveness of our system.

Future work will focus on designing signal waveforms to achieve high-accuracy ranging and robust communication, paving the way for a joint communication, localization, and mapping system.

## Figures and Tables

**Table 1:** The absolute trajectory error (ATE, meters) for each sequence, where bold represents the optimal result.

	A-LOAM	LIO-SAM	FAST-LIO2	RLI-SLAM (3 anc)	RLI-SLAM (2 anc)	RLI-SLAM (1 anc)	RLI-SLAM (rand)	RLI-SLAM (0 anc)	RLI-SLAM (3 anc w/o LCD)	RLI-SLAM (2 anc w/o LCD)	RLI-SLAM (1 anc w/o LCD)	RLI-SLAM (rand w/o LCD)
*hall_01*	0.204	0.205	0.219	0.107	0.162	0.172	0.154	0.172	**0.092**	0.161	0.170	0.153
*hall_02*	0.271	0.369	0.505	0.132	0.354	0.378	0.398	0.421	**0.124**	0.347	0.352	0.312
*door_01*	0.266	0.232	0.399	**0.189**	0.273	0.294	0.258	0.312	0.257	0.297	0.303	0.293
*door_02*	0.220	0.177	0.311	**0.138**	0.273	0.282	0.192	0.291	0.175	0.320	0.391	0.298
*gata_01*	0.566	0.184	0.164	**0.116**	0.135	0.145	0.258	0.312	0.257	0.297	0.303	0.293
*gata_02*	0.420	0.494	0.276	**0.274**	0.279	0.281	0.292	0.291	0.285	0.298	0.276	0.278
*gata_03*	0.170	0.101	0.201	**0.098**	0.113	0.128	0.139	0.121	0.115	0.120	0.191	0.198
*street_01*	6.355	35.790	281.430	**2.805**	3.403	3.223	11.721	12.391	4.750	7.379	10.127	8.912
*street_02*	2.625	3.045	2.240	**1.549**	2.855	3.291	4.468	4.821	2.000	3.198	3.215	2.986
*street_04*	3.153	0.822	6.087	**0.185**	0.303	0.270	0.354	0.791	0.261	0.311	0.391	0.397
*street_08*	3.185	0.596	0.501	**0.178**	0.349	0.388	0.302	0.323	0.287	0.441	0.539	0.571

Note: “anc” stands for the number of UWB anchor points, “rand” indicates a random number of UWB anchor points, and “LCD” indicates loop closure detection.

**Table 2:** The absolute trajectory error (ATE, meters) for each sequence, where bold represents the optimal result.

	A-LOAM	LIO-SAM	FAST-LIO2	VIRAL-Fusion	RLI-SLAM	RLI-SLAM (w/o LCD)
*eee_01*	0.212	0.075	0.131	0.060	**0.054**	0.063
*eee_02*	0.199	0.069	0.124	0.058	**0.047**	0.054
*eee_03*	0.148	0.101	0.163	**0.037**	0.069	0.082
*nya_01*	0.077	0.076	0.122	0.051	**0.046**	0.055
*nya_02*	0.091	0.090	0.142	**0.043**	0.058	0.082
*nya_03*	0.080	0.137	0.144	0.052	**0.046**	0.052
*sbs_01*	0.203	0.089	0.142	0.048	**0.047**	0.058
*sbs_02*	0.091	0.083	0.140	0.062	**0.049**	0.056
*sbs_03*	0.363	0.054	0.133	0.054	**0.048**	0.056

Note: “LCD” indicates loop closure detection.

**Table 3:** Breakdown of processing time.

Module	Time (ms)
The Inertial Sensor Preprocessing	2.62
UWB Optimization	0.21
Point Cloud Feature Processing	6.93
State Optimization Estimation	28.72
Building Point Cloud Maps	1.38
Loop Detection	1.71
Total Time	41.57

## Data Availability

Data are contained within the article.
